# The Transition from Occupational Safety and Health (OSH) Interventions to OSH Outcomes: An Empirical Analysis of Mechanisms and Contextual Factors within Small and Medium-Sized Enterprises

**DOI:** 10.3390/ijerph15081621

**Published:** 2018-07-31

**Authors:** Guido J. L. Micheli, Enrico Cagno, Antonio Calabrese

**Affiliations:** Department of Management, Economics and Industrial Engineering, Politecnico di Milano, Piazza Leonardo da Vinci, 32, 20133 Milan, Italy; guido.micheli@polimi.it (G.J.L.M.); antonio.calabrese@polimi.it (A.C.)

**Keywords:** OSH, interventions, mechanisms, contextual factors, barriers, drivers, SMEs

## Abstract

Many Occupational Safety and Health (OSH) interventions have proven to be effective only under controlled conditions; during the implementation in practice, the interventions may not work as expected, especially in small and medium-sized enterprises (SMEs). SMEs are affected by different contextual factors than larger enterprises and these factors can influence the outcome of the OSH programs. Three different phases of an OSH intervention (design, implementation, and control) have been considered. The aim of this research is to understand what are the mechanisms by which an OSH intervention works or does not work as expected, together with barriers and drivers, and the related contextual factors. The research was designed following multiple case study research, which enables an in depth understanding of the intervention process and the identification of the most relevant factors for OSH. Data were collected through interviews with owner-managers or OSH managers of SMEs. Finally, the data were analysed through an analytical research framework that enabled the identification of the main mechanisms and contextual factors for the interventions that had an expected outcome and for those which had an unexpected outcome.

## 1. Introduction

Occupational Safety and Health (OSH) is concerned with protecting the safety, health and welfare of people engaged in work or in employment. It aims at the adaptation of working environment to workers in all occupations for the promoting and the maintenance of the highest degree of physical, mental, and social wellbeing.

This need is driven by social and economic considerations. According to the Labour Force Survey, using data from 2007, 3.2% of the workforce in the EU-27 (approximately 6.9 million persons) reported an accident at work in the past 12 months. Data from the European Statistics on Accidents at Work (ESAW) showed 2.9% of the workers had an accident at work with more than three days of sickness or absence and 5580 workers died in a fatal accident in 2007. For occupational diseases, according to the LFS (European Union Labour Force Survey) using data from 2007, 8.6% of the workforce in the EU-27 (approximately 23 million persons) reported a work-related health problem in the past 12 months. In total, 2.1% of the persons had two or more work-related health problems (Eurostat European Commission, 2010; elaborated from source: i.Stat). Moreover, work-related injuries and illnesses cost the global economy an estimated $1,250,000 million U.S. dollars [1].

When it comes to small and medium-sized enterprises (SMEs, i.e., companies with less than 250 employees), these figures are even more staggering. The European Agency (EU-OSHA) acknowledges for the “old” EU-15 member-states (data refer to 2011) nearly 19 million SMEs, employing about 75 million people; contribute to approximately 82% of all occupational injuries, rising to about 90% of fatal accidents. Thus, it is plain that the role of SMEs is particularly important in Europe (Table 1), and especially in Italy, where SMEs are unconditioned economical protagonists. With reference to the National Institute of Statistics (data 2015, elaborated from source: i.Stat), in Italy there are 4,334,419 SMEs (99.9% of the enterprises/establishments), where 4,108,750 operate in manufacturing, construction and services (accordingly with the NACE classification), equivalent to 94.7% of the total and 94.8% of SMEs (data 2015, elaborated from source: i.Stat). The added value produced by SMEs in Italy amounts to 500 billion euros, which is 70% of the total. In terms of employment, the part played by these enterprises is even more relevant: 79.4% of employees work in SMEs (Census 2011; elaborated from source: i.Stat).

However, the OSH conditions in SMEs are very often poorer than in the larger enterprises. Small and medium-sized enterprises usually have a higher accident rate (in Italy it is not unusual that more than 90% of the reported OSH injuries correspond to SMEs) and worse consequences. The causes of this situation, can be found in the scarcity of human, economic, and technological resources [2,3]. Further studies (as reviewed by [4]) underlined that the low level of occurrence of accidents and injuries a SMEs can experience lowers risk perception, alters approach to risk control, and changes management priorities. Thus, only large severity accidents can have a long-term impact on an OSH management system, but it can often be too late to intervene [5]. Table 1 shows data referred to SMEs and large enterprises in EU-28.

The improvement of safety conditions, especially in SMEs, is a very important task and requires practitioners dedicate human, technological, and economic resources to OSH, and that these resources are used effectively [6,7]. In a few words, the improvement of OSH conditions within the enterprises requires the implementation of effective OSH interventions [7,8,9].

An OSH intervention is defined as an attempt to improve safety and health conditions in workplaces by means of targeted activities and initiatives. Such activities include changes in work organization and working conditions, engineering activities for modification and installation of plant and equipment, training, and behavioural changes. Interventions can occur at different levels.

However, nowadays, when enterprises try to change and to improve their work-environment by targeted activities and procedures, the result is often not the one expected. The lack of effectiveness of safety interventions is one of the major problems affecting companies, especially SMEs, in Italy.

Therefore, the overall aim of this research is understanding the mechanisms by which an OSH intervention at the workplace level works or does not work as expected, by adopting the procedure and related taxonomies of mechanisms and contextual factors recently developed by Masi et al. [10]. By means of a questionnaire, several interviews were conducted in enterprises to reach a clear view on interventions and on the related outcomes. The adoption of the structured approach proposed by [10] claims for better supporting practitioners in the organisation all their knowledge, information and a data in a more systematic way, helping them to better understand the phenomena that take place and thus to improve their capacity of finding new and more effective solutions.

The paper is structured as follows: Section 2 introduces the conceptual research framework; Section 3 presents the literature review; Section 4 describes the methodology of the research; Section 5 summarizes the results; Section 6 discusses the results and; finally, Section 7 draws the conclusions.

This article can be considered as a small part of the SESAME project (EUOSHA-PRU/2014/FC-02), which aims to contribute an informed EU-wide analysis of current knowledge concerning the nature and experience of risk to health and safety and its occurrence in work in small and micro enterprises, in this case also in medium enterprises. It aims to give a better understanding of how risk is most effectively addressed in relation to these enterprises, while taking proper account of their structure, operation, and context. In so doing, it further aims to inform discourse on future policy development in this important area while at the same time helping identify important gaps in current understandings.

## 2. Problem Setting

The lack of effectiveness of OSH activities, especially in SMEs, has led researchers to propose complex frameworks of intervention to explain why it can work or it cannot work as expected.

In the past, the literature was focused on interventions in general, concentrating mostly on a better understanding of companies’ safety programs. The findings of this research were not fully exploitable by enterprises because of two main reasons: the first is that they were not able to provide a comprehensive picture embracing all the factors related to the safety performance. Such a picture is essential, since establishing a proper intervention policy starts from a systematic identification of all the OSH-related factors and interactions describing the company’s safety performance. The second reason, is that it is possible to study the relationship between two factors independently from the context in which this relationship takes place only in situations where the conditions of the context are assumed constant and, like in almost all the cases in the industrial practice, it is necessary to include in the model an adequate number of mediating factors representing the influence of the context on the relationship between the two main factors considered in the study [5].

Nowadays, researchers emphasize the need to consider the broader context in which the intervention process takes place, arguing the probability of success of an intervention is crucially dependent on the context affecting the implementation [11,12,13]. In the view of the authors, a realistic analysis is an approach that can provide a stepping-stone for the development of an analytical framework for the analysis of the intervention considering the contextual factors.

The basic idea of the realist analysis is to study how, for whom, and under what circumstances an OSH program works. Thus, the key concept is that a program needs to have a mechanism that will make a target group in a specific context make changes resulting in the desired outcome. The model is simple: Mechanisms + Context = Outcome, a model of causality. During the development of a program, it is possible to find specific reactions. These reactions follow certain logics and rules, which depend on the context, but also have similarities from case to case. Pawson and Tilley [14] suggested this similarity could be understood as an interaction between mechanisms and context. Interventions are not presumed to have causal powers in themselves, instead context and mechanisms are seen as the factors that initiate or trigger the causal relationships, so, the actual outcome of an intervention varies depending on the intervention, the context, the mechanisms and the interplay between these factors and can be categorized as positive, negative, expected, or unexpected [13]. Chang and Moleh [15], in accident analysis, and Kim and Jung [16], in human reliability analysis, proposed comprehensive taxonomies of behavioural mechanisms and contextual factors, but they cannot adapt to OSH interventions because of some specific features such as the complexity of the interplay among worker behaviours, intervention specific contextual factors, and longer time horizon.

Cagno et al. [5] proposed a model for the design of OSH interventions that takes into account the contextual factors and the behavioural mechanisms affecting the implementation of the OSH interventions. The proposed model enabled the identification and the understanding of the main likely interventions patterns, thanks to a structured view, a proper level of detail and operationalization, and a simple representation of the overall view that can be immediately understood and used by SMEs’ managers.

Nevertheless, as suggested by Masi et al. [10], the ideal proposed studies for the analysis of the interventions are often not fully exploitable by OSH practitioners during the design of OSH intervention because they do not guide practitioners into a systematic and structured fashion to identify the mechanisms and the contextual factors relevant for a particular OSH intervention. The frameworks limited themselves to providing a generic definition of mechanisms and contextual factors, assuming practitioners will be autonomously able to identify them on the basis of their skills and previous experience. The problem is practitioners could be unable to properly identify specific mechanisms and contextual factors, since the practitioners decision-making processes can at best be described as governed by bounded rationality, in which heuristic and prior experience play a large role.

**Figure 1:**
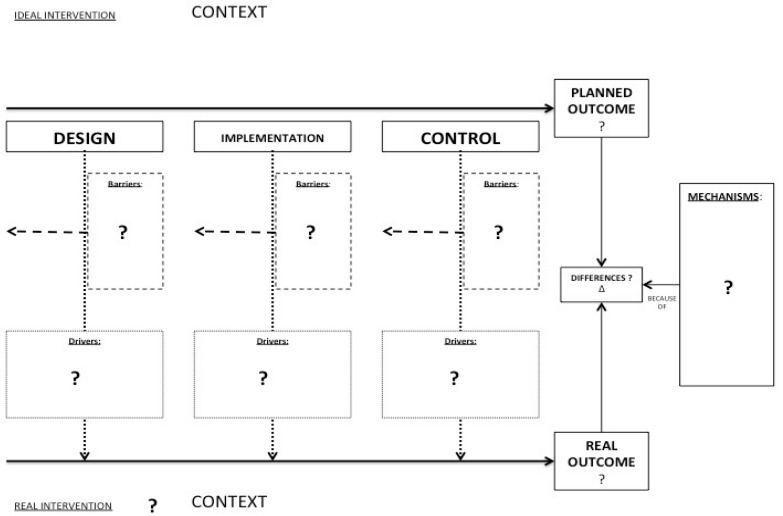
Conceptual research framework (“?” is the object of the investigation; “Δ” is the difference between the planned outcome and the real outcome).

Based on the literature review, this research aims at understanding what are the mechanisms by which an OSH intervention works or does not work as expected, together with barriers and drivers, and the related contextual factors, as depicted in the conceptual research Framework in Figure 1.

The framework was structured as illustrated in Figure 1 because it aims at the identification of the mechanisms in an easy way, focusing on the possible differences between the planned outcome and the real outcome and taking in consideration the contextual factors, including drivers and barriers that can act at different phases of the OSH intervention, namely design, implementation, and control. As noticed by Masi and Cagno [17], the three phases are conceptually and temporally related; however, although related, some drivers or barriers (that are specific contextual factors) can be more critical in specific phases of the intervention process. Drivers and barriers, as the literature review will show, are contextual factors not directly involved in workplace safety interventions. Nevertheless, they significantly affect, either fostering or hindering, the outcome of the interventions.

## 3. Literature Review

All the elements included in the conceptual research framework have been described thanks to a literature review that enabled a focus on:

- Mechanisms
- Contextual factors
- Drivers and barriers

The aim of this section is to present a comprehensive portrait of available knowledge about OSH interventions from a review of the research and literature concerning the main pillars (i.e., mechanisms, contextual factors, drivers, and barriers) to set up the fundamental questions. The search of the literature was made using two of the main international database on-line (namely, Google Scholar and Scopus), using as initial key words “OSH” combined with “intervention”, “mechanisms”, and “contextual factors”. Thereafter, an iterative process (snowball in terms of references, as well as “cited by” search) and a final check hand-search within the top recurring journals (i.e., including more than 3 relevant papers) was performed.

By screening the title, the abstract and the key words, the articles were selected to be fully read; then, the most representative ones were coded in a large database. The phase of coding was extended during the reading to new information, for example “drivers” and “barriers”. Moreover, articles about SMEs were sought because the focus of this research is mostly on SMEs. The search has concentrated on works published in English (since it tends to represent both the majority and the most authoritative sources in international resources databases). The search has been limited to material published from 1984 onwards. The literature review was used as a point of departure for more detailed fields of studies to follow.

### 3.1. Mechanisms

A clear and unique definition of mechanisms does not exist. Pawson et al. [18] defined mechanism as “the engine of explanation in realist analysis… We rely on mechanisms to tell us why interconnections should occur… Mechanisms explain causal relations by describing the “powers” inherent in a system”. Pedersen et al. [13] defined mechanisms as relevant personal characteristics of key actors or interpersonal relations between them. For Masi et al. [10], mechanisms are a “mental state of key actors that are triggered by a program, that vary with changes in the context, and that produce a change in the performance or in the behaviour of the workers”.

In this research, mechanisms are considered as the elements that can explain why an intervention works or does not work as expected, but, despite their importance, the literature about them is quite young. The theoretical contributions are limited.

Hale et al. [19] present an early description of the mechanisms lying behind an intervention that might tell why the project was successful or not. These authors concluded interventions that bring about constructive dialogue between shop floor and line management, provide motivation to line managers, and strengthen the monitoring and learning loops in the safety management system, appeared more successful.

Pedersen et al. [13], inspired by the realist analysis, where mechanisms are one of the fundamental pillars, considered the implementation of an OSH intervention dependent on the mechanisms promoting social change and dependent on the contextual factors that enable or disable these mechanisms. In a later work, Hasle et al. [20] pointed out the influence of context on mechanisms; mechanisms and context together have an impact on the outcome in terms of improvement (or otherwise) in the working environment. The study was concentrated on the analysis of an intervention aimed at the reduction of the risk of musculoskeletal disorders. In Legg et al. [21] mechanisms are considered to understand how they influence the OSH programs of governments. Only the study of Masi et al. [10] gives it a complete taxonomy of mechanisms, which was used in this research to identify the results (Table 2). Their research made a practical example of the relation between a mechanism and a contextual factor; it detected that perception of the familiarity with the situation was influenced by the contextual factor layout of the plant.

### 3.2. Contextual Factors

Contextual factors play a decisive role in the design in the implementation and in the control of an OSH intervention. Nowadays a new stream of research is growing in which several models have been proposed to characterize the intervention process through the description of the context [13]. Pedersen et al. [13] have been among the first researchers who maintained that the same intervention can have different outcome related to different contextual factors. The factors of external and internal context are then decisive for the outcome of the interventions, and therefore they must be considered during design, implementation, and evaluation [10].

Many OSH interventions have proven to be effective under controlled conditions, but their implementation in practice is often difficult and interventions may not work as expected, especially in SMEs [10].

Masi et al. [10] proposed a definition of contextual factors, referring to them as “factors that are not directly related to the performance or to behaviour of the workers, but that are expected to influence the performance or the behaviour substantially”. So, the context can limit the effectiveness of a program, enabling or disabling mechanisms. The specific role of context is to create the conditions to put in practice a correct OSH program. Less human, economic, and technological resources are the most common contextual factors described in the literature [2,3,22,23]. Some other researchers focused on the factors related to the deficiencies in the organizational process, management, and compliance with regulation [5,21,24,25]. However, management in SMEs is typically informal and the process to manage operational activities are not very structured and formalized. Especially in SMEs, many owner-managers often do production work and have close social relationships with their employee, so they find it difficult and uncomfortable to exert their authority [10].

These studies cannot support practitioners in the design of more effective OSH interventions because of a limited understanding of the role of factors within the more general implementation process and the way in which factors interact. Existing studies proposed factors that can play a key role in the implementation of intervention, such as the resistance of workers to change their behaviour or the management commitment and attitudes towards OSH. However, it is not clear how to promote management commitment or how to overcome the resistance of workers to change their behaviour. In the same way, it is not clear whether these factors are equally important in different contextual conditions.

- The most commonly perceived contextual factors were:
- Less human, economic and technological resources
- Low level of management and training skills
- Lack of knowledge of company’s risks
- Cost of using consultants

Table 3 schematizes the contextual factors used for the analysis in this research.

#### Drivers and Barriers

Drivers and barriers can be considered a spin-off of contextual factors. They identify all those contextual factors not directly involved in workplace safety interventions but significantly affecting, respectively fostering or hindering, the outcome of interventions [26]. Walker and Tait [27] investigated the effectiveness of an approach used in the UK, designed to help small enterprises to set up and operate a simple health and safety management system, discovering several drivers helping enterprises in to implement an effective safety management system. Whysall et al. [28] explored the process of implementing interventions to tackle occupational ill health, in particular the facilitators and the barriers involved in implementing such interventions. The factors cited as key barriers and facilitators included the resistance of workers to change their behaviour, gaining managerial commitment, and managers’ general attitudes towards health and safety.

Cagno et al. [29] gave a first picture of drivers for the OSH interventions in SMEs. This study is fundamental because it investigated the drivers in the function of the phases of the intervention process. For example, the design seems mainly driven by knowledge while the implementation seems mainly driven by economic resources. The authors proposed a classification of drivers (Table 4), considering all the contributions from the literature, and used it on a sample of 58 SMEs. The most commonly perceived drivers were:

- External support of consultants
- Availability of knowledge of effective interventions
- Collaborations with associations and network of companies
- ICT tools supporting OSH interventions
- Reduction of insurance premium by the national compensation authorities

Barriers are those factors hindering the proper design, implementation, and evaluation of OSH interventions [26]. There is little consensus on how barriers should be understood, how important they are in different contexts, and how they can hinder OSH interventions. Champoux and Brun [23] determined some key barriers: costs, paperwork, lack of training, priority to production, lack of time, lack of staff, employee attitudes, employee demands, planning difficulties, and profitability of investment in preventions. Walker and Tait [27] studied the organization and the implementation of a simple health and safety management system. The main results were: (a) the presence and appropriateness of the health and safety policy statement and the risk assessment; (b) the presence and the appropriateness of documentation for training, maintenance, first aid treatments and accidents; (c) the presence and the appropriateness of training standards, maintenance standards, and general standard of premises and welfare facilities, and finally; (d) other information such as manager attitudes and particular problems encountered. The inappropriateness of these factors considered in the interviews were seen as a barrier for OSH in the enterprises.

Hasle and Limborg [30] focused on the difficulty to comply with legal requirements and on the lack of resources allocated to OSH interventions. Masi and Cagno [17] introduced a classification scheme to put order to and classify all the factors interrupting the process; the macro categories are person related, organization related, regulation related, resources related. 

Little empirical investigation exists about the importance of barriers in the enterprises and the understanding whether other firm-related features such as the phase of the intervention process, or the firm’s size, or firm’s industrial sector can affect barriers. The taxonomy created by Masi and Cagno [17] was used for the analysis of data (Table 5).

The most commonly perceived barriers were:

- Regulation problem (bureaucracy and stringent legal requirements)
- Resources problems (lack of time and lack of economic resources)
- Information problems (absent or ineffective communication, absent or ineffective information, lack of awareness of OSH relevance by workers, lack of technical support by control authorities and lack of guidelines)

## 4. Research Issues/Problems/Gaps

The OSH literature is sectorial and fragmented. Occupational Safety and Health researchers have specific sectorial competences and orientation/background, which can be either technical, psychological, or behavioural, so these researchers often limit their research to their competences and orientation. Moreover, one can detect a lack of theoretical materials about OSH in SMEs. As a consequence, still a lack of a comprehensive framework exists, that accounts for all the factors analysed. 

**Figure 2:**
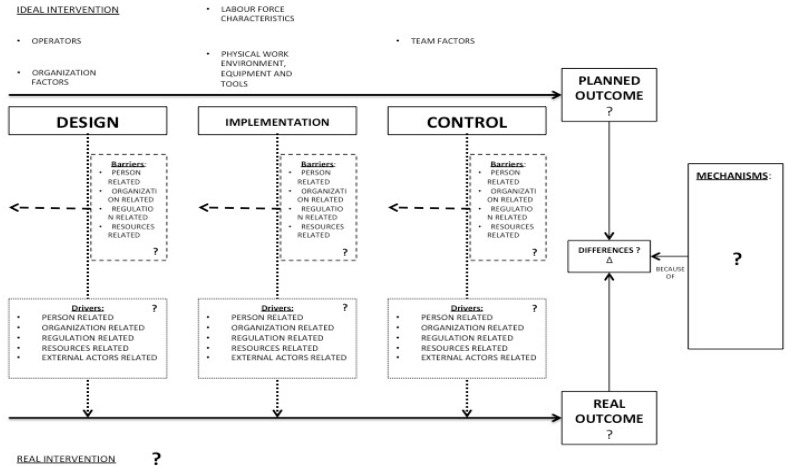
The analytical research framework (“?” is the object of the investigation; “Δ” is the difference between the planned outcome and the real outcome).

Additionally, in the reviewed studies it is not clear which are the factors and in which phases of the intervention process they are more relevant. Other proposed studies are often too theoretical; practitioners instead need procedures that are simply, easy to use, and systematic. For these reasons, the proposed analytical research framework (Figure 2) tries to overcome the limitations of the literature by presenting a complete and systematic view of the phenomenon that:

- Is SME specific
- Considers the phases of the intervention
- Studies the problem from the workplace point of view, thereby being less theoretical

This study tries to investigate the “HOWs” and the “WHYs” with an in-depth analysis of the intervention process, understanding why there could be a difference between the planned outcome and the real one. Specifically, the objectives of the research are:

1. Understanding which the mechanisms are and in which context they act to determine the outcome of an OSH intervention
2. Researching in which phases of the intervention, the factors (drivers and barriers) are more influential.

The study of the contextual factors, the drivers and the barriers and the knowledge of the related mechanisms associated to different contexts enable practitioners to change and modify the design of safety policies in advance. This will allow them to have a successful implementation of safety programs and a higher control of the events. The analysis of the data is easier and faster and it is possible to contextualize the mechanisms in a graphic way, pointing out drivers and barriers for the different phases of the intervention process.

## 5. Methodology

This research project performed a multiple-case study to get a comprehensive description of OSH interventions including mechanisms and contextual factors, in the light of the suggestion of Yin [31], who recommended the use of case study when the research focuses on the “how” and “why” of a problem. This strategy of research provides a rich in-depth understanding of the intervention process and the identification of the crucial factors or characteristics.

This research considered a case study to be more appropriate than experiments, surveys, and histories to analyse OSH interventions because, following the definition given by [31], contemporary phenomena are investigated in their real context with an in-depth analysis, the boundary conditions between the investigated case and the context are not so clear and evident and, many different sources have been used to collect data.

The unit of analysis is a generic OSH intervention. Mechanisms occurring for a single intervention could be different from case to case and the only way to have a complete view on the mechanisms is by having a complete description of the interventions. In this research, 58 OSH interventions were analysed, coming from 43 SMEs of the industrial sector in Lombardia, Italy. Only industrial sectors were selected (thus, excluding agriculture, services, and so on), because the OSH interventions considered could also be technical and techno-organizational, not only organizational. The companies were contacted by means of professional associations.

Data for the research have been collected through interviews using a questionnaire. The interview used a predefined questionnaire composed of two main sections, context, and intervention.

For the first section, the clusters of information were two. The section “context” was dedicated to the characterization of the respondent (in particular, seniority in the specific activity, training, competences, human relations, OSH interest, intern or external role were investigated; it was also specified if the queries were only for employers, only for HSE managers or for both) and to the identity record of the company (in particular, sector, kind of production, number of employees, company size, certification, OSH organization and OSH importance were investigated). The same reasoning was made for the section “intervention”, which was dedicated to the description of the case. The main topics were: intervention kind, “HOWs” & “WHYs”, development, drivers, barriers, outcome and cost.

The interviews were conducted with 40 employers, 11 of which were also HSE managers in their enterprise; in nine cases, when the employer was not available for the interview, the internal HSE managers were asked to answer the questions instead of the owner-manager. Employers and HSE managers were considered the most appropriate for this kind of research because, in the enterprise, they are the ones who have a complete view on design, implementation, and control of the intervention. Moreover, when it was possible, supplementary interviews with workers and external consultants were conducted to increase validity and create triangulation.

Each interview lasted no more than 30 min, considering in some cases a period of informal discussion after the formal registered interview. Interviews were tape recorded, with the agreement of the participants. All recorded material was fully transcribed, verbatim. Then a report of each interview was written and sent to the different enterprises to check the correctness of the information.

One enterprise with three cases was used as pilot. The pilot tested a first version of the questionnaire to verify the consistency of the questions. After the review, another interview was made in the pilot enterprise to get the missing information.

The data were coded in a database according to the analytical framework. A single case analysis was then performed to identify the main mechanisms for each case.

## 6. Results

The data have been analysed according to the kind of intervention, the results of the intervention, and the reasons for acting. Of the 58 studied cases:

- 36 were organizational OSH interventions
- 19 were technical OSH interventions
- three were techno-organizational OSH interventions

It was specified if the interventions were mandatory or voluntary. To have a clear sample, the cases were first checked to verify if they had been implemented after some serious work-related injury. The attention for safety after work-related accidents increases and the data for those cases could be distorted. For this reason, two cases have been not considered in the analysis.

### 6.1. Organizational Interventions

An organizational intervention refers to activities whose aim is the functional coordination of OSH actions, namely educating and training activities as well as safety-related policies and procedures. In this research, 36 organizational interventions were analysed, 12 of which had an unexpected outcome and 24 of which had an expected outcome.

#### 6.1.1. Unexpected Outcome

An organizational intervention with “unexpected outcome” refers to activities of education, training, or policies and procedures in which the intended result was not completely achieved.

- The interventions were:
- Mandatory training courses: educational activities conducted due to law requirements
- Voluntary training courses: educational activities organized to make the workers more aware of safety
- Design and implementation of voluntary procedures

**Figure 3:**
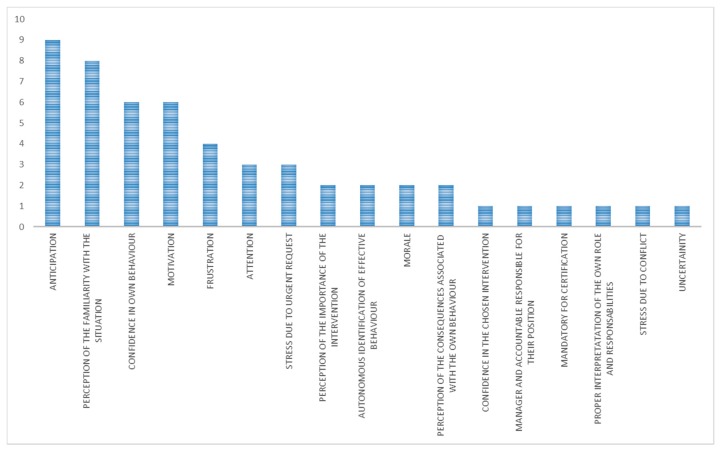
Main mechanisms (in terms of number of occurrences) in organizational interventions with unexpected outcome.

**Figure 4:**
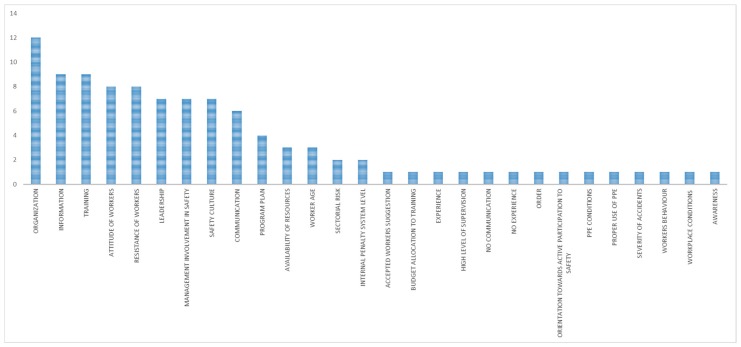
Main contextual factors (in terms of number of occurrences) in organizational interventions with unexpected outcome.

The main mechanisms identified for the whole category “organizational interventions with unexpected outcome” were anticipation and perception of the familiarity with the situation (Figure 3). The main contextual factors (Figure 4) were work and tasks organization, information, training, attitudes of workers, resistance of workers, leadership, management involvement in safety, safety culture, and communication.

The main driver observed in the design phase and in the implementation phase was the external support of consultants, whereas the main barriers observed in the design phase and in the implementation phase were the lack of awareness of OSH relevance by workers and the excess of external bureaucratic requirements, especially for the mandatory interventions.

#### 6.1.2. Expected Outcome

An organizational intervention with “expected outcome” refers to activities of education, training, or policies and procedures in which the intended result was completely achieved.

These interventions could be:

- Mandatory training courses
- Voluntary training courses
- Design and implementation of mandatory procedures
- Design and implementation of voluntary procedures

**Figure 5:**
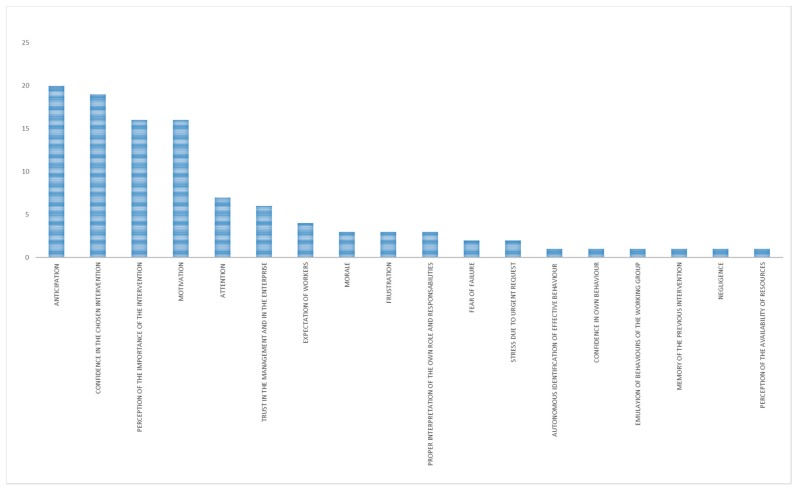
Main mechanisms (in terms of number of occurrences) in organizational interventions with expected outcome.

The main mechanisms identified for the category “organizational interventions with expected outcome” were (Figure 5) anticipation, confidence in the chosen intervention, perception of the importance of the intervention, motivation, and awareness of the OSH relevance by workers.

**Figure 6:**
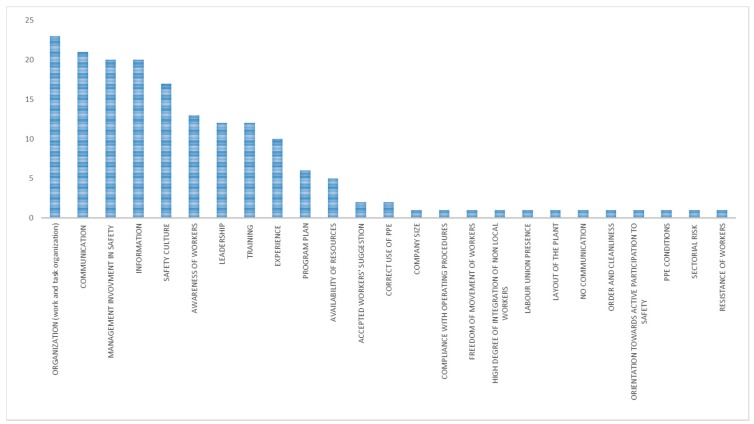
Main contextual factors (in terms of number of occurrences) in organizational interventions with expected outcome.

The main contextual factors (Figure 6) were work and tasks organization, communication, management involvement in safety, information, safety culture, leadership, and training.

The main driver observed in the design phase and in the implementation phase was the external support of consultants, whereas the main barrier observed in the design phase and in the implementation phase was the excess of external bureaucratic requirements.

### 6.2. Technical Interventions

A technical intervention refers to engineering solutions that decrease the probability of a worker engaging in at-risk behaviour. All the technical cases analysed had an expected outcome.

The technical interventions could be:

- Mandatory technical interventions
- Voluntary technical interventions

**Figure 7:**
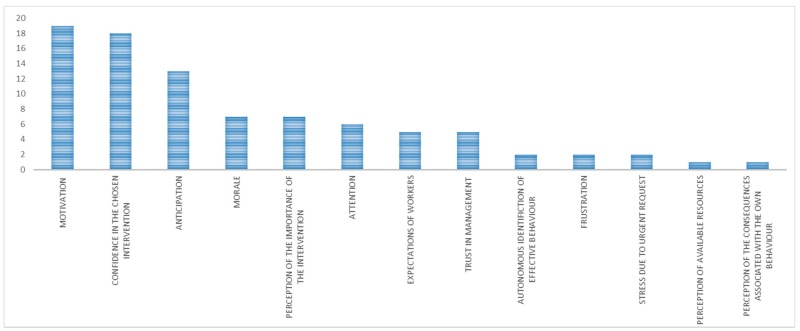
Main mechanisms (in terms of number of occurrences) in technical interventions with expected outcome.

The main mechanisms identified for this category (Figure 7) were motivation, confidence in the chosen intervention, and anticipation.

**Figure 8:**
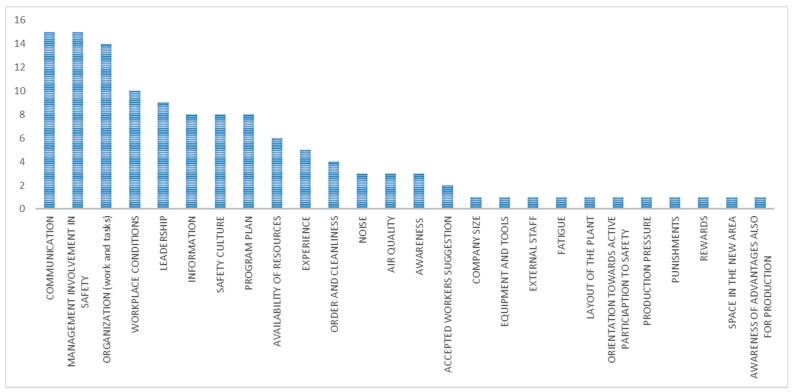
Main contextual factors (in terms of number of occurrences) in technical interventions with expected outcome.

The main contextual factors (Figure 8) were communication, management involvement in safety, organisation, workplace conditions, and leadership.

The main driver observed in the design phase and in the implementation phase was the external support of consultants, whereas the barrier most mentioned (but only in seven cases out of 19) was the lack of economic resources in the design and implementation phase.

### 6.3. Techno-Organizational Interventions

**Figure 9:**
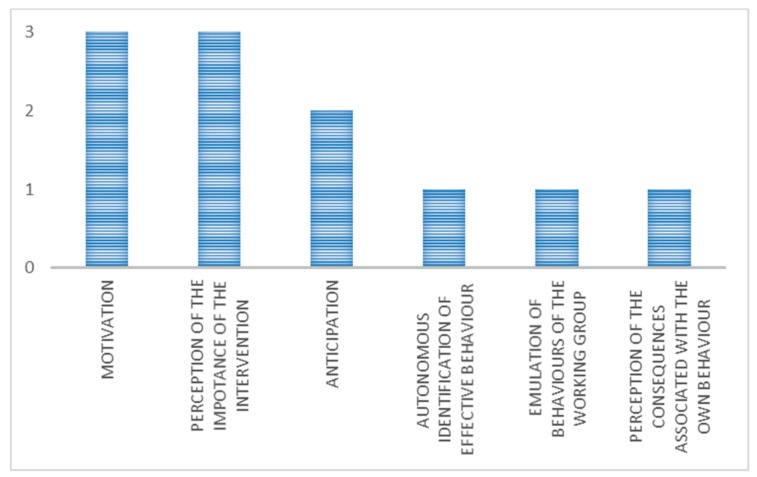
Main mechanisms (in terms of number of occurrences) in techno-organizational interventions with expected outcome.

A techno-organizational intervention refers to an engineering solution combined with a new procedure. Only three cases belonged to this category. The main mechanisms (Figure 9), identified for all the three interventions, were motivation, perception of the importance of the intervention, and anticipation.

**Figure 10:**
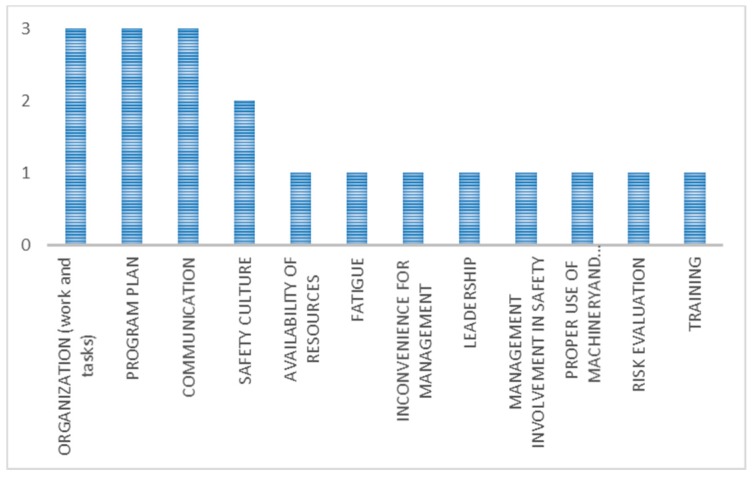
Main contextual factors (in terms of number of occurrences) in techno-organizational interventions with expected outcome.

The main contextual factors (Figure 10) were work and tasks organization, program plan, and communication.

Only in the design phase, a driver was observed: bonus, award, and incentives from INAIL.

The barriers were mainly economic-related.

## 7. Discussion

**Figure 11:**
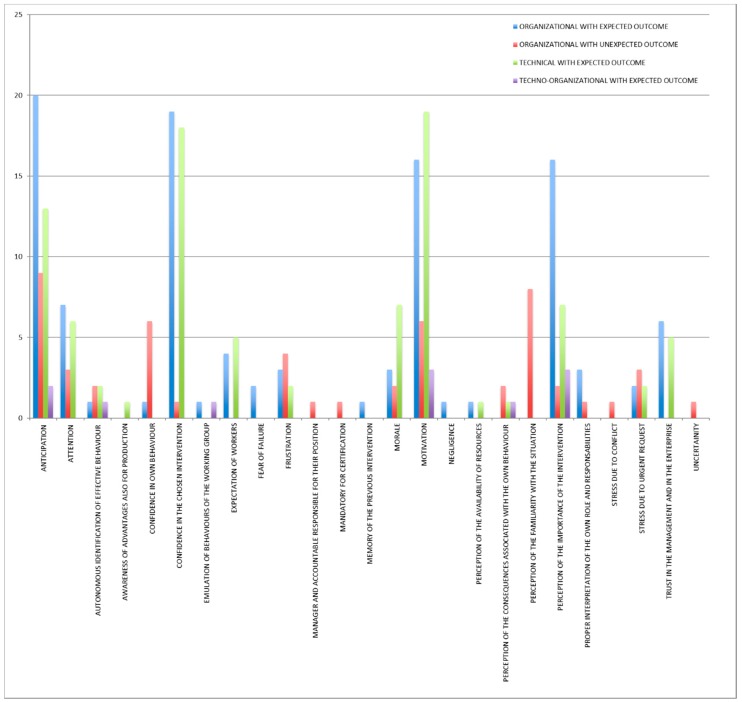
Comparison of mechanisms (in terms of number of occurrences).

The results of this research enabled a clear view on the mechanisms of the OSH interventions. Precise and different mechanisms were identified for the cases with unexpected outcome and the ones with expected outcome. When interventions did not work as expected, the prevailing mechanisms identified were (in more than half of the cases) (Figure 11):

- Anticipation
- Perception of the familiarity with the situation

**Figure 12:**
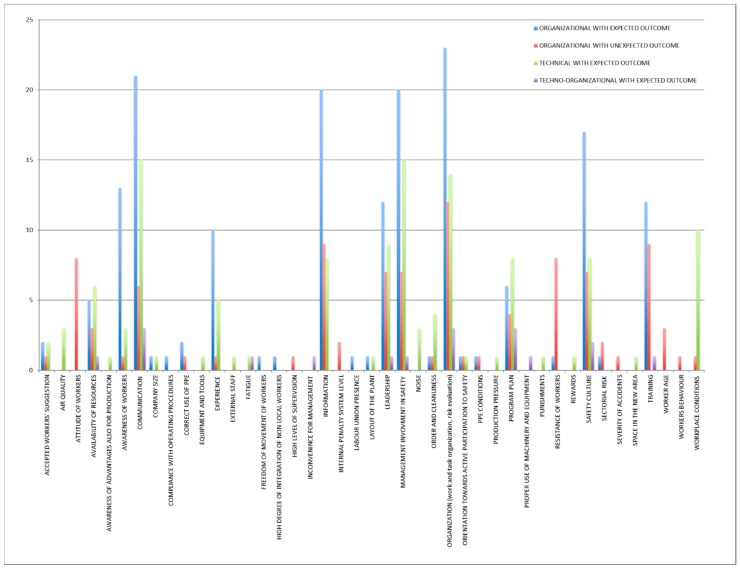
Comparison of contextual factors (in terms of number of occurrences).

The main contextual factors affecting an intervention with unexpected outcome were (Figure 12):

- Work and tasks organization
- Information
- Training
- Resistance of workers
- Leadership
- Management involvement in safety
- Safety culture
- Attitude of workers

Most of these factors have already been considered in the literature review, not only as contextual factors, but also as drivers or barriers. Whysall et al. [28] considered the resistance of workers as one of the key barriers in the OSH interventions. He also added that *management involvement in safety* was an important driver.

One of the most commonly perceived drivers in the literature (e.g., [29]) was ICT tools and information. Moreover, in the theoretical review, *communication* has always been considered with positive meaning; Whysall et al. [28] referred to it as a way for the improvement of the OSH conditions. In this research, communication emerged as not only an important factor for the intervention with “expected outcome”, but also for the ones with “unexpected outcome”. However, communication is not enough to motivate workers to change their attitudes and habits regarding OSH. Often interventions did not work as expected when there is the combination of *perception of the familiarity with the situation* with the factor *resistance of workers* and *attitudes of workers* and the barrier in the implementation phase *lack of awareness of OSH relevance by workers*.

The mechanism *perception of the familiarity with the situation* has already been analysed in the paper by [4], where it was linked with the contextual factor *layout of the plant*. In this present research, that relationship was not observed.

Another important contextual factor was *training*. Employers and external consultants agreed the only way to make workers to feel responsible for their actions is by training them. In the cases analysed, the employers were aware of worker indifference to OSH. So most of them decided to implement safety courses; this is why training was listed among the contextual factors. It takes some time and several training courses to see improvement.

In the literature, another one of the most perceived contextual factors was *low level of management and training skills*, but in this research it was observed that, not only the interventions with unexpected outcome, but also the interventions with an expected outcome were characterized by the contextual factors *management involvement in safety* and *leadership*. Moreover, only in two of the 12 cases with unexpected outcome did the employers not attend a safety course. Among the 40 interviewed employers, 26 attended a safety course, 30 employers declared high/good training skills, and at least 28 reported a high/good preparation in the OSH field.

For some cases, a link was observed between the mechanisms *frustration* and *stress due to urgent request* and the factor *lack of program plan*.

The prevailing mechanisms observed in interventions with expected outcome were:

- Anticipation
- Confidence in the chosen intervention
- Motivation
- Perception of the importance of the intervention
- Trust in management and in the enterprise

The important observed factors were:

- Communication
- Experience
- Information
- Leadership
- Management involvement in safety
- Work and tasks organization
- Program plan
- Safety culture
- Workplace conditions
- Awareness of workers

For the organizational interventions with expected outcome, the main factors observed were awareness of OSH relevance by workers combined with confidence in the chosen intervention, motivation and perception of the importance of the interventions. The confidence in the chosen interventions was often related to management involvement in safety.

The high level skills of management can explain why the contextual factor *work and tasks organization* was always present with the mechanisms *anticipation*, both in the interventions with expected outcome and in the one with unexpected outcome. To avoid work-related injuries, companies implemented in advance or made changes in procedures, machines and tools, as well as organizing work and tasks of employees.

*Workplace conditions* are particularly important for the technical interventions. Many of them were implemented to improve safety conditions in the enterprises by modifying workplace conditions. Moreover, for the technical interventions, *motivation* and *confidence in the chosen interventions* as mechanisms, combined also with another mechanism, *morale,* were important.

For drivers and barriers, common considerations can be made for the interventions with expected and unexpected outcome together. In most of the analysed cases, *external support of consultants* was fundamental in the design and in the implementation phases, but this driver has already been underlined in the literature, which also mentioned the cost of consultant as a factor. No employer in this research has indicated external consultant as a cost.

As a barrier, most enterprises indicated the *excess of external bureaucratic requirements*. Only the technical interventions indicated as barriers in the design and in the implementation phases the *lack of economic resources*, the *lack of time,* and the *lack of technical resources*. These were typical barriers from the literature (e.g., [10]). In this research, however, the lack of these elements was not so widespread as expected from the literature review.

The control phase was often characterized, in the few cases which talked about control, by *fear of sanctions by control authorities*. This driver is strictly related to the main barriers observed in most of the interventions, namely the excess of external bureaucratic requirements.

During the analysis of data, differences between SMEs were investigated, but the only distinction that emerged was OSH certification ISO 18001, more common in the medium-sized enterprises. This was not relevant for the results of the research.

If, in the future, Italian legislation moves towards internal HSE managers, this will cause a loss of information, especially in small enterprises. Most small companies rely on external consultants. Even if sometimes the owner-manager is the one who decides on the interventions to implement, the HSE manager is the one to promote the most important interventions, checks for new law requirements and updates the documents. In the interviewed companies, 28 of 43 enterprises had a HSE manager as an external worker.

It is necessary to underline the importance of the mechanism *perception of the importance of one’s own role and responsibilities* because it can make the difference. The influence of this mechanism was mentioned not only by employers, but also by the external consultants. It can help to make workers feel more aware about OSH relevance.

## 8. Conclusions

The study proved there are interesting combinations of contextual factors and mechanisms that will enable a better understanding of OSH interventions. For example, the link between the factor *work and tasks organization* and the mechanism *anticipation* or *perception of the familiarity with the situation* with *attitude of workers* and *resistance of workers* is key in all cases. Furthermore, employers of SMEs can consider some general advice to improve OSH conditions. Workers must be made to feel responsible for their actions and they should be more aware about OSH relevance. This is a difficult task, but with focused training activities, workers can be helped to learn the importance of OSH. Moreover, when interventions have unexpected outcome, often there is a tendency to blame the management only, thus reducing in practice the true importance of the workers that should continue to give importance to OSH and should also pay more attention at the control phase. The main drivers and barriers were identified only for the design and the implementation phase. The control phase was often omitted, or not so much taken in consideration. Legislators also have an important consideration. Many companies said that *the excess of external bureaucratic requirements* was one of the most problematic barriers. Legislators should focus on and understand the different needs of SMEs to reduce bureaucratic requirements and improve OSH conditions.

As for a limitation of this study, its research was restricted to 58 cases, nonetheless it is very challenging and hard to reach out to SMEs because the “safety” topic is a delicate one. Moreover, this research was limited to the industrial sector, where the major risks exist. In light of all considerations, future research should:

- Extend the sample
- Extend the research to other sectors
- Take in consideration the workers’ perspectives

A broader sample could compare more data to find similarities and differences in the combinations of contextual factors and mechanisms. Future researches could extend the study to other sectors and analyse them separately to understand the specific mechanisms for each sector. Different risks can affect different sectors.

The relationship between the health, safety, and welfare of workers and the role played by social dialogue in helping to determine workers’ experience of health and safety are topics not so much discussed in OSH research, especially in SMEs. The experiences related to workers are too often invisible and it is fundamental to describe and improve the knowledge about them to understand the weaknesses and to explore further gaps in OSH. It is necessary to understand why an intervention cannot be effective from the workers’ point of view. New scenarios can be examined and these evaluations can contribute to the building of new policies that more aware of the needs of workers.

The results were achieved by adopting the taxonomies and the procedure introduced by Masi et al. [10], revealing how a structured approach can help practitioners in the organisation all their knowledge, information and a data in a more systematic way, even if limiting their creativity to some extent. This structured approach cannot be claimed only by realist analysis, but the latter provides the opportunity to shed the light on the different layers of the phenomena [12], so better catching their real essence and thus be more effective in understanding and design solutions [13].

It also seems likely the most profitable approach for future empirical studies would be to adopt a “mixed-method” strategy for data collection, taking into consideration not only managers’ experience, but also workers.

The improvement of the effectiveness of the OSH interventions is enabled by knowledge of the different elements affecting the OSH program. A better investigation of the combination between mechanisms and contextual factors is necessary in further research, using as point of departure the result of this present study.

## Figures and Tables

**Table 1:** SMEs (small and medium-sized enterprises) and large enterprises: number of enterprises, employment and value added in EU-28 in 2013 (elaborated from Eurostat, National Statistical Offices).

	Micro	Small	Medium	SMEs	Large	Total
**Number of enterprises**						
Number	19,969,338	1,378,374	223,648	21,571,360	43,517	21,614,877
%	92.4%	6.4%	1.0%	99.8%	0.2%	100.0%
**Employment**						
Number	38,629,012	27,353,660	22,860,792	88,843,464	44,053,576	132,897,040
%	29.1%	20.6%	17.2%	66.9%	33.1%	100.0%
**Value added at factor cost**						
Million Euros	1,362,336	1,147,885	1,156,558	3,666,779	2,643,795	6,310,574
%	21.6%	18.2%	18.3%	58.1%	41.9%	100.0%

**Table 2:** Mechanisms.

Class	Mechanism
**Temporary cognitive states**	Memory of the previous interventions
	Anticipation
	Autonomous identification of effective behaviours
	Perception of the importance of the intervention
	Perception of the consequences associated to behaviours
	Expectation of workers
	Perception of familiarity with the situation
	Proper interpretation of the own role and responsibilities
	Perception of the available resources
	Perception of the complexity of intervention
	Emulation of behaviours of working group
	Resistance of workers
**Psychological states**	Motivation
	Morale
	Confidence in own behaviours
	Confidence in the chosen intervention
	Trust in management and in the enterprise
	Fear of failure
	Stress due to urgent request
	Stress due to conflict
	Frustration
	Uncertainty
	Attention

**Table 3:** Contextual factors.

Class	Sub-Class	Factor
**Operators**	**Physical factors**	Fatigue
		Physical abilities
	**Cognitive factors**	Attitude of worker
		Experience
		Knowledge
		Resistance of workers
		Skills
		Training
		Workers’ behaviour
		Workers’ awareness of OSH relevance
	**Age**	Workers’ age
	**Participation**	Accepted workers’ suggestion
		External staff
		Orientation towards active participation to safety
**Physical work environment, equipment and tools**	**Physical work environment**	Illumination
		Noise
		Air quality
		Temperature and Humidity
		Freedom of movement
		Freedom of communication
		Layout of the plant
		Workplace conditions
		Sectorial risk
		Company size
		Space available
		Severity of accidents
		Order and cleanliness
	**PPE**	Correct use of PPE and conditions of PPE
	**Equipment and tools**	Availability
		Proper use of machinery and equipment
		Quality
**Organization factors**	**Management and policy**	Availability of resources
		Inconvenience for management
		Information
		Internal penalty system
		Labour union presence
		Level of supervision
		Management involvement in safety
		Plant policy
		Production pressure
		Program plan
		Punishments
		Rewards
		Safety culture
**Organization factors**	**Management and policy**	Training
		Work and task organization
	**Tasks**	Task related difficulties
		Other difficulties
	**Procedures**	Compliance with operating procedures
		Availability
		Quality
	**Production**	Awareness of advantages and production pressure
**Team factors**	**Features of the team**	Cohesiveness
		Composition
		Coordination
		Degree of integration of non-local workers
	**Communication**	Availability
		Quality
	**Leadership**	Leadership

**Table 4:** Drivers.

Driver
Rewards, bonuses and awards from the company
Monetary incentives from central or local control authorities
Reduction of fees for deserving companies
Reduction of the insurance premium by the national compensation authority
Reduction in bank lending rates
Insurance benefits
Sanctions by control authorities
Behaviour of unions
Tailor made legislation for SMEs
External support in the OSH management by control authorities
External support of consultants
Collaborations with associations and networks of companies
Training programs for SMEs
Communication tools
ICT tools
Knowledge of effective interventions
Collaboration with other stakeholders (customers, contractors, and suppliers)

**Table 5:** Barriers.

Kind	Level	Barrier
**External**	**Government**	Stringent legal requirements
		Bureaucracy
	**Regulators, associations**	Lack of technical support by control authorities
		Behaviour of trade union
		Difficulties in the interaction with external agencies
		Lack of guidelines
	**Intermediaries**	Lack of technical support by consultants
**Internal**	**Management**	Systematically wrong behaviour of management
		Management not adequately skilled
		Lack of knowledge of the criticalities of company by management
		Lack of knowledge of the profitability of the interventions by management
	**Staff**	Systematically wrong behaviour of personnel
		Personnel not adequately skilled
		Lack of knowledge of the criticalities of companies by workers
		Lack of awareness of OSH policy
	**Organization**	Inadequate OSH policy
		Scarce involvement of personnel in OSH activities
		Lack of time
**Internal**	**Organization**	Lack of economic resources
		Inadequacy of the organization
		Absent or ineffective communication
		Absent or ineffective information
		Prioritization of production over safety
		Difficulty in planning the OSH activities
		Difficulty in obtaining authorizations by management
	**Technology**	Lack of technical resources
		Absent or ineffective information collection system
